# Healthcare Access for a Diverse Population with Schizophrenia Following the Onset of the COVID-19 Pandemic

**DOI:** 10.1007/s10597-023-01105-1

**Published:** 2023-05-18

**Authors:** Marcela Horvitz-Lennon, Emily Leckman-Westin, Molly Finnerty, Junghye Jeong, Jeannette Tsuei, Katya Zelevinsky, Qingxian Chen, Sharon-Lise T. Normand

**Affiliations:** 1 RAND Corporation, 20 Park Plaza, Suite 920, Boston, MA 02116 USA; 2 Department of Psychiatry, Cambridge Health Alliance and Harvard Medical School, 1493 Cambridge Street, Cambridge, MA 02139 USA; 3 Office of Mental Health, New York State Department of Health, 44 Holland Avenue, Albany, NY 12229 USA; 4 Department of Epidemiology and Biostatistics, School of Public Health, University at Albany, State University of New York, 1 University Pl, Rensselaer, NY 12144 USA; 5 RAND Corporation, 1776 Main Street, Santa Monica, CA 90407 USA; 6 Department of Health Care Policy, Harvard Medical School, 180 Longwood Avenue, Boston, MA 02115 USA; 7 Department of Biostatistics, Harvard School of Public Health, 677 Huntington Ave, Boston, MA 02115 USA

**Keywords:** Schizophrenia, Access, Race and ethnicity, Equity, COVID-19

## Abstract

COVID-19 has had a disproportionate impact on the most disadvantaged members of society, including minorities and those with disabling chronic illnesses such as schizophrenia. We examined the pandemic’s impacts among New York State’s Medicaid beneficiaries with schizophrenia in the immediate post-pandemic surge period, with a focus on equity of access to critical healthcare. We compared changes in utilization of key behavioral health outpatient services and inpatient services for life-threatening conditions between the pre-pandemic and surge periods for White and non-White beneficiaries. We found racial and ethnic differences across all outcomes, with most differences stable over time. The exception was pneumonia admissions—while no differences existed in the pre-pandemic period, Black and Latinx beneficiaries were less likely than Whites to be hospitalized in the surge period despite minorities’ heavier COVID-19 disease burden. The emergence of racial and ethnic differences in access to scarce life-preserving healthcare may hold lessons for future crises.

**Supplementary Information:**

The online version contains supplementary material available at 10.1007/s10597-023-01105-1.

## Introduction

COVID-19 has had a disproportionate morbidity and mortality impact on the most disadvantaged members of society, including racial and ethnic minorities and those with disabling chronic illnesses including serious mental illnesses (Chowkwanyun & Reed, 2020; NYC Health, 2020; Webb Hooper et al., 2020). People with schizophrenia are more likely to experience poorer COVID-19 outcomes because of their higher rates of obesity, diabetes, and other pre-existing physical health conditions, and their profound social disadvantage, including higher rates of homelessness (Chen et al., 2020; Druss, 2020; Fond et al., 2021; Jegede et al., 2020; Lee et al., 2020; Murphy et al., 2021; Tsai & Wilson, 2020; Wang et al., 2021). In fact, a study conducted in a New York City healthcare system during the early phase of the pandemic found that schizophrenia was a more significant risk factor for COVID-19-related mortality than heart failure, cancer, and other serious medical and psychiatric conditions (Nemani et al., 2021).

Evaluating access to critical healthcare during the early phase of the COVID-19 pandemic remains an important scientific endeavor as disrupted access may be hard to restore; moreover, treatment discontinuity may lead to illness exacerbations or unrelated declines in physical health, further adding to these individuals’ large burden of disease (Druss, 2020; Kozloff et al., 2020). Longstanding drivers of racial and ethnic healthcare disparities may have created more access barriers for minority groups including non-Latinx Black, hereafter Black, and Latinx individuals relative to their non-Latinx White, hereafter White, counterparts.

The evidence on access to healthcare by individuals with schizophrenia during the early phase of the COVID-19 pandemic is limited, particularly regarding equity of access. Small studies found no changes or increases in outpatient mental healthcare utilization for individuals with a broad spectrum of diagnostic severity (Lynch et al., 2020; Miu et al., 2020; Yang et al., 2020). However, a national study of adult Medicare beneficiaries with serious mental illnesses including schizophrenia found reductions in routine outpatient and acute utilization of care for mental health or substance use disorders, hereafter, behavioral healthcare (Busch et al., 2022). A large national study found that people with serious mental illnesses were more likely to be hospitalized and die following COVID-19 diagnosis than individuals without serious mental illnesses (Murphy et al., 2021). We are not aware of research that has examined this population’s early post-pandemic experiences with community-based behavioral health services typically financed by Medicaid or non-COVID-19 related physical healthcare. Moreover, little is known on differences by race and ethnicity on health service utilization beyond evidence that relative to White Medicare beneficiaries, Blacks were less likely to receive behavioral health outpatient (BHO) services (Busch et al., 2022).

We conducted a study to examine the impacts of the COVID-19 pandemic in New York State’s diverse adult Medicaid beneficiary population with schizophrenia with a focus on racial and ethnic equity of access to critical healthcare in the immediate post-pandemic surge period. Our goal was to fill a key gap in the evidence that may provide valuable lessons for future responses to public health emergencies.

## Methods

### Study Cohort and Data Source

We included New York State Medicaid beneficiaries aged 18–64 years who during the study period had at least 1 month of enrollment and a diagnosis of schizophrenia as indicated by at least one inpatient or outpatient claim with schizophrenia ICD-10 codes (F20, F25) in any position. We created an open cohort, meaning beneficiaries could enter or exit anytime between 1 year preceding the onset of the COVID-19 pandemic in the state (pre-pandemic period or pre-period: March 7, 2019–March 6, 2020), inclusive of a pneumonia season (March 7, 2019–May 15, 2019), and a 70-day early phase following its onset (post-pandemic “surge” period or post-period: March 7, 2020–May 15, 2020). We selected March 7, 2020 as the date of pandemic onset because the state declared a state-of-emergency to contain the spread of the virus on this date (New York State, 2020). Consistent with other research (Gupta et al., 2020; Ziedan et al., 2020), we selected May 15, 2020 as the end of the surge period because this is the date of the termination of the “New York State on PAUSE” executive order that required statewide closure of non-essential businesses and “stay at home” orders among other restrictions. The first calendar date the beneficiary meets inclusion criteria is denoted denote as their index eligibility date. We excluded beneficiaries with dual Medicare–Medicaid coverage because our focus is on beneficiaries only having Medicaid coverage given differences in policies between the Medicare and Medicaid programs. We also excluded those lacking information on their county of residence (< 1.4%). Our sole data source was Medicaid data maintained by the Office of Mental Health (OMH), New York State’s mental health authority (see Supplementary Section “Analytic Plan” for further details on our data source).

### Outcomes and Independent Variables

We constructed six binary outcome measures, all but one assessed daily, selected based on their critical importance for this population. Individuals receiving care for schizophrenia need close outpatient monitoring of symptoms and treatment response, often need high-intensity community-based services, have a high suicide risk, and are also at high risk for serious medical comorbidities. Three measures captured utilization of BHO care: (1) *routine BHO care* and (2) *antipsychotic drug prescription fills,* both of which individuals receiving care for schizophrenia should access regularly, and (3) *high-intensity BHO care*, a composite measure that captured *monthly* utilization of any of three team-based specialty services typically reserved for more seriously ill patients: Assertive Community Treatment (ACT); Personalized Recovery Oriented Services (PROS), a psychosocial rehabilitation program; or behavioral health Home and Community-Based Services. The other three measures captured inpatient utilization for life-threatening emergencies: (4) *psychiatric admissions for suicidality* (hereafter, suicide-related admissions); (5) *admissions for cardiometabolic emergencies* (hereafter, cardiometabolic admissions), a composite measure that captured admissions for cardiovascular disorders or diabetes mellitus; and (6) *pneumonia admissions* assessed during the pneumonia season, which as of March 2020, included COVID-19 pneumonia.

Our main independent variables of interest were beneficiary race and ethnicity (Whites, non-Latinx Black, Latinx, Asian/Other, and unknown race and ethnicity), time (count of days or months set to 0 at the initiation of the pandemic), pandemic period (pre- and post-pandemic), and their interaction. Other variables included beneficiary sex, age (categorical: 18–24, 25–44, 45–64), county of residence (N = 62), and whether Medicaid-eligible through Supplemental Security Income (SSI), which we employed as a health status indicator given that SSI eligibility requires demonstration of disability. See Supplementary Section “Analytic Plan” for further details on our outcome and independent variables.

### Statistical Analysis

Standardized mean differences of person and person-day characteristics between the post and pre-pandemic periods using the standard deviation in the pre-period were computed to assess balance. Standardized differences larger than 0.10 or smaller than − 0.10 suggest imbalances between the two periods. We estimated unadjusted rates of service utilization expressed as mean and standard errors (SEs) per 100,000 person-days or, for high-intensity BHO care, person-months. We determined the mean post-pandemic versus pre-pandemic differences (post–pre-pandemic differences) and 95% confidence intervals (CIs), and for the two composite measures (high-intensity BHO care, cardiometabolic admissions), we also provide results for the individual components.

For adjusted analyses, we utilized logistic regression models at the beneficiary-day (month) level, modeling the probability of occurrence of the outcome on the day (month), and estimated separate models for each of the six outcomes. All models included race and ethnicity (White as reference), time (day or month), sex (female as reference), SSI, age groups (45–64 years as reference), pandemic period (pre-period as reference), and county fixed effects (New York City as reference), with possible pairwise interaction terms including race and ethnicity, time, and county. Day (or month) was parameterized using linear and quadratic terms to capture non-linear trends. Models that included interactions of race and ethnicity with day (month), pandemic period, and county of residence were also estimated (see Supplementary Section “Analytic Plan” for further details on statistical models, and Supplementary Table 1 showing estimated regression coefficients). A final model for each outcome was selected using the model with the smallest Akaike Information Criterion (Forster & Sober, 2011). Odds ratios (ORs) and 95% CIs for differences between racial and ethnic minority groups and Whites were computed. We did not adjust for multiple observations contributed by each beneficiary and CIs were not adjusted for multiplicity of estimation.

Data were analyzed using SAS version 9.4.

The study was reviewed and approved by the authors’ IRBs.

## Results

### Characteristics of the Study Cohort

The numbers of beneficiaries meeting study eligibility in the pre-pandemic and post-pandemic periods were comparable (67,184 and 63,369, respectively). Due to the different durations of the pre- and post-pandemic periods, these individuals contributed different numbers of person-days, 22,976,921 and 4,383,072, respectively (Table 1). Over 75% of individuals were enrolled for the entire 14.5-month study period. The mean (SE) number of days enrolled per beneficiary is 406.5 (0.29).

**Table 1:** Characteristics of the Study Cohort (Persons and Person-days)

Characteristic	Pre-pandemic March 7, 2019–March 6, 2020		Post-pandemic March 7, 2020–May 15, 2020		Standardized Mean Differences	
	Persons	Person-Days	Persons	Person-Days	Persons	Person-Days
Number	67,184	22,976,921	63,369	4,383,072		
Female, n (%)	26,173 (39.0%)	9,041,810 (39.4%)	24,818 (39.2%)	1,719,169 (39.2%)	0.0042	− 0.0026
Age Group, n (%)						
18–24	6863 (10.2%)	2,277,333 (9.9%)	6354 (10.0%)	438,705 (10.0%)	− 0.0062	0.0033
25–44	30,174 (44.9%)	10,158,267 (44.2%)	28,283 (44.6%)	1,955,283 (44.6%)	− 0.0056	0.0080
45–64	30,147 (44.9%)	10,541,321 (45.9%)	28,732 (45.3%)	1,989,084 (45.4%)	0.0094	− 0.0100
Mean/Median (SE)	41.8/42.0 (0.05)	42.1/42.0 (0.00)	41.9/42.0 (0.05)	42.0/42.0 (0.01)	0.0105	− 0.0112
Race and Ethnicity, n (%)						
Black	26,112 (38.9%)	8,971,082 (39.0%)	24,674 (38.9%)	1,706,607 (38.9%)	0.0014	− 0.0022
Latinx	12,694 (18.9%)	4,487,750 (19.5%)	12,237 (19.3%)	848,332 (19.4%)	0.0106	− 0.0045
White	16,399 (24.4%)	5,645,007 (24.6%)	15,523 (24.5%)	1,075,964 (24.5%)	0.0020	− 0.0005
Asian/Other	4545 (6.8%)	1,543,980 (6.7%)	4270 (6.7%)	296,123 (6.8%)	− 0.0011	0.0015
Unknown	7434 (11.1%)	2,329,102 (10.1%)	6665 (10.5%)	456,046 (10.4%)	− 0.0174	0.0089
SSI, n (%)	38,075 (56.7%)	13,632,584 (59.3%)	36,271 (57.2%)	2,523,092 (57.6%)	0.0114	− 0.0360

Pre- and Post-Pandemic Periods

*Source* Authors’ analysis of New York State Medicaid data (March 7, 2019–May 15, 2020)

All standardized mean differences were <|0.01|, suggesting good balance in race and ethnicity and other patient characteristics, between the pre and post-pandemic periods (Table 1). While Blacks represented over one-third of the cohort (38.9 and 39%, persons and person-days respectively) and Latinx individuals represented close to a fifth (18.9 and 19.5%, respectively), Whites represented about a quarter of the cohort. We were unable to determine race and ethnicity for about 10% of the cohort. Slightly more than half of the cohort had become eligible because of their disability (SSI) (Table 1).

The small percentage of beneficiaries (1.4%) who were excluded due to not having county information tended to be older, more male, and Black, and less likely to be White or have SSI (see Supplementary Table 2 showing characteristics of beneficiaries included compared to those excluded).

### Unadjusted Findings

Differences in utilization rates between the pre- and post-pandemic periods were only observed for high-intensity BHO care and pneumonia admissions. While rates increased for both outcomes, as expected, the increase for pneumonia admissions was large: pre-pandemic mean (SE) was 3.3 (0.3) per day and a mean (95% CI) post–pre-pandemic difference of 12.2 (9.8, 14.5) per day (see Supplementary Table 3 showing unadjusted utilization rates).

**Fig. 1:**
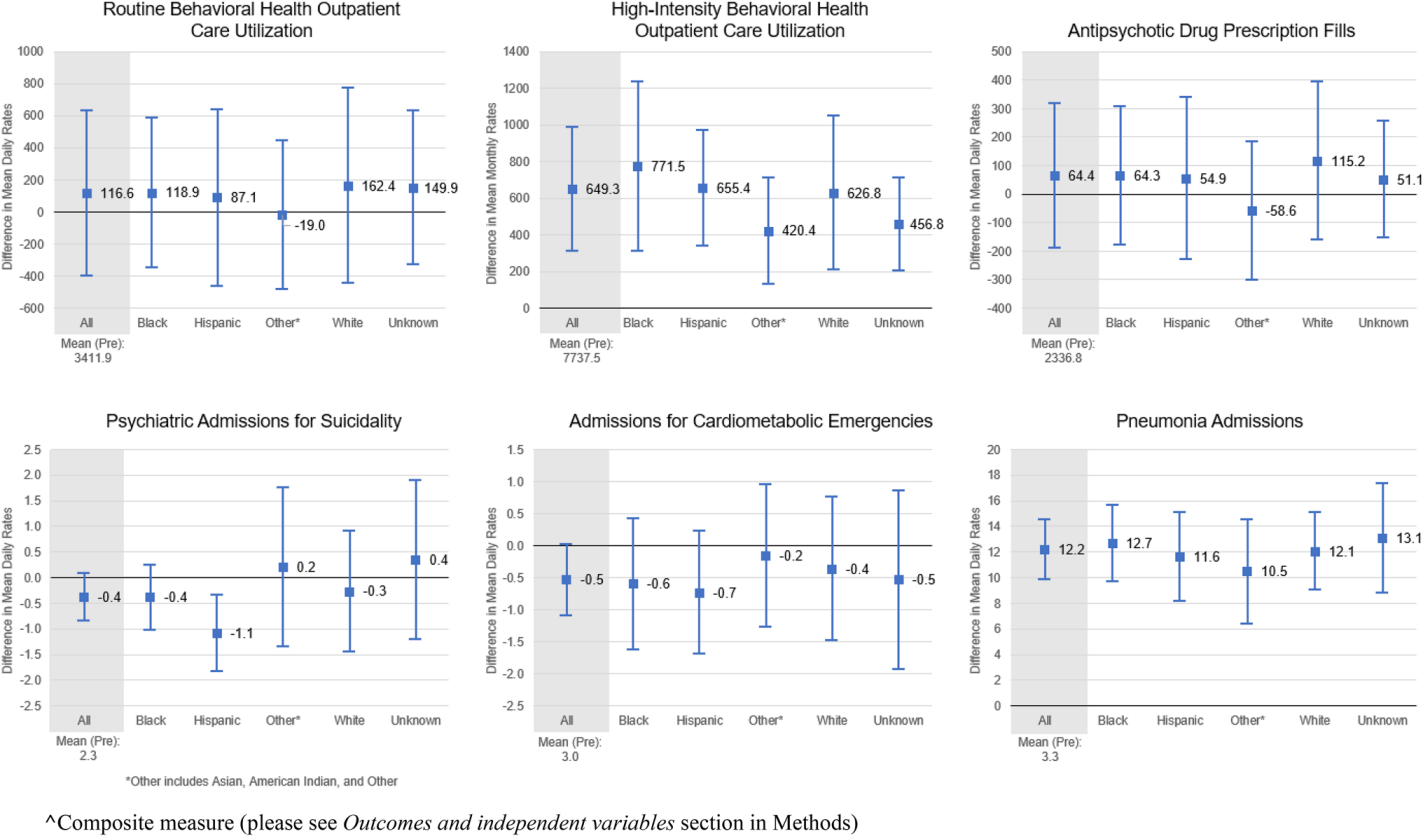
Unadjusted Utilization Rates per 100,000 person-days (or person-months). Mean (95% CI) Differences between the Post-pandemic and Pre-pandemic periods (Post–Pre Differences), by Race and Ethnicity

Unadjusted results suggested some differences in post–pre-pandemic differences by race and ethnicity for high-intensity BHO care, *antipsychotic drug prescription* fills, and suicide-related admissions (Fig. 1).

### Adjusted Findings

Differences in utilization by race and ethnicity across all outcomes were observed, but the temporal patterns varied across outcomes.

**Fig. 2:**
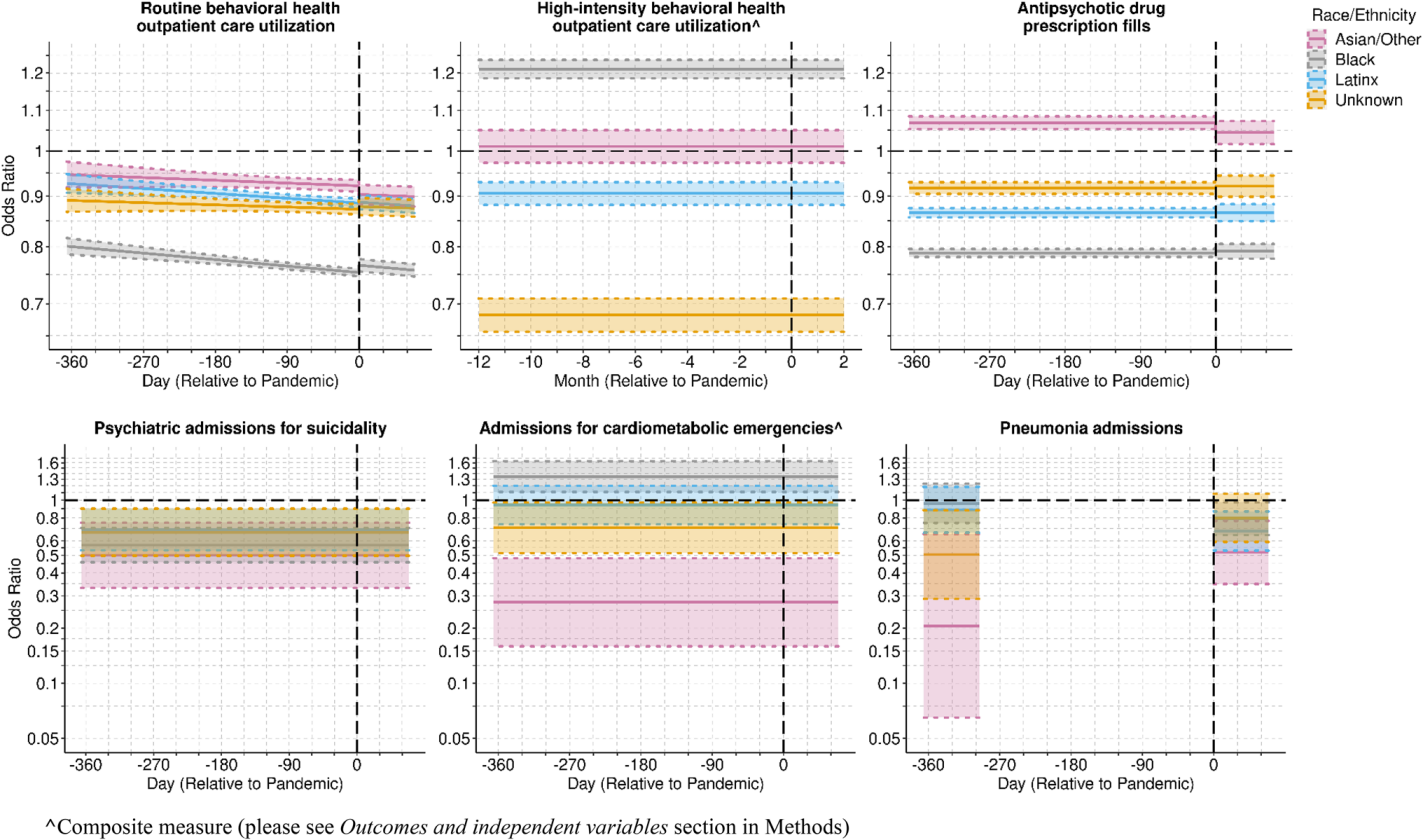
Adjusted Odds of Healthcare Utilization. Odds Ratios by Time, for Non-White groups versus Whites and Pointwise 95% Confidence Intervals

Racial and ethnic differences in high-intensity BHO care, suicide-related admissions, and cardiometabolic admissions did not change over time (Table 2a; Fig. 2). Throughout the study period, Black beneficiaries had higher odds of high-intensity BHO care than Whites (OR = 1.2; 95% CI = 1.19, 1.24), while the opposite was true for Latinx beneficiaries (OR = 0.91; 95% CI = 0.88, 0.93). All non-White groups had lower odds of suicide-related admissions, with Asian/Other beneficiaries having the lowest odds (OR = 0.50; 95% CI = 0.33, 0.75). Blacks were 1.3 times as likely as Whites to be admitted for cardiometabolic emergencies (OR = 1.35; 95% CI = 1.11, 1.64), with Latinx beneficiaries having comparable odds, and Asian/Other beneficiaries were about one-quarter as likely (OR = 0.28; 95% CI = 0.16–0.48).

**Table 2:** Adjusted Odds of Healthcare Utilization by Race and Ethnicity

(a) Associations not changing with time or pandemic period. Reference group is White						
Racial and ethnic group	High-Intensity Behavioral Health Outpatient Care^		Psychiatric Admissions for Suicidality		Admissions for Cardiometabolic Emergencies^	
	Odds Ratio	95% CI	Odds Ratio	95% CI	Odds Ratio	95% CI
Black	1.21	(1.185–1.237)	0.568	(0.458–0.705)	1.347	(1.109–1.635)
Latinx	0.906	(0.882–0.93)	0.691	(0.532–0.898)	0.941	(0.739–1.199)
Asian/Other	1.011	(0.973–1.05)	0.500	(0.332–0.754)	0.277	(0.159–0.482)
Unknown	0.682	(0.656–0.709)	0.668	(0.497–0.898)	0.707	(0.514–0.971)

(b) Associations changing with time or pandemic period. Reference group is White				
Racial and ethnic group	Antipsychotic Drug Prescription Fills		Pneumonia Admissions	
	Odds Ratio	95% CI	Odds Ratio	95% CI
Black versus White				
Pre-pandemic	0.788	(0.781, 0.796)	0.962	(0.751, 1.231)
Post-pandemic	0.792	(0.778, 0.805)	0.792	(0.645, 0.972)
Latinx versus White				
Pre-pandemic	0.866	(0.857, 0.875)	0.886	(0.665, 1.182)
Post-pandemic	0.866	(0.849, 0.884)	0.679	(0.531, 0.868)
Asian/Other versus White				
Pre-pandemic	1.068	(1.053, 1.084)	0.206	(0.065, 0.652)
Post-pandemic	1.044	(1.017, 1.073)	0.519	(0.348, 0.772)
Unknown versus White				
Pre-pandemic	0.917	(0.905, 0.930)	0.505	(0.289, 0.881)
Post-pandemic	0.921	(0.899, 0.945)	0.801	(0.592, 1.084)

Odds Ratio and 95% Confidence Interval (CI)

Odds Ratios for Routine behavioral health outpatient care changed daily—they are only reported graphically

^Composite measure (please see *Outcomes and independent variables* section in Methods)

*Source* Authors’ analysis of New York State Medicaid data (March 7, 2019–May 15, 2020)

While racial and ethnic differences in utilization changed over time for the other outcomes, most changes were minimal (Table 2b; Fig. 2). The odds of having routine BHO care were lower for non-White groups than Whites throughout the study period, with Blacks having the lowest odds. Although the odds decreased daily for non-White groups, thus widening the gap, a small change in the rate of change was observed in the surge period, with Blacks having a small increase in this utilization relative to the pre-pandemic period.

Non-White groups also had different odds than Whites of having *antipsychotic drug prescription* fills in both periods, with negligible changes over time. Black, Latinx, and beneficiaries of Unknown race and ethnicity had lower odds, but the odds of Asian/Other beneficiaries were higher.

Patterns of pneumonia admissions did change over time. While the odds for Black and Latinx beneficiaries relative to Whites in the pre-pandemic period did not differ, they decreased relative to Whites in the surge period, e.g., Latinx beneficiaries were 0.7 times as likely as Whites (OR = 0.68; 95% CI = 0.53, 0.87). On the other hand, a pre-pandemic difference for those of Unknown race and ethnicity dissipated, and it became smaller for Asian/Other beneficiaries (pre-period: OR = 0.21; 95% CI = 0.065, 0.65; post-period: OR = 0.52; 95% CI = 0.35, 0.77).

## Discussion

Our study of the early access effects of the COVID-19 pandemic among adult Medicaid beneficiaries with schizophrenia in New York State found that utilization of critical services and differences by race and ethnicity remained largely unchanged following the onset of the pandemic. This constancy is noteworthy given the severity of the pandemic in the state and how much it strained its healthcare system. However, like others (Horvitz-Lennon et al., 2009, 2013), we found that racial and ethnic minorities had lower utilization of services that are a mainstay of schizophrenia care (routine BHO care and AP drugs) both in the pre-pandemic and the surge periods. Moreover, we found a new difference in pneumonia admissions emerging during the COVID-19 surge period between Whites and the state’s largest minority groups.

Because our only health status indicator was evidence of disability based on receipt of SSI benefits, the observed racial and ethnic differences in utilization of high-intensity BHO care and admissions for life-threatening conditions may have been driven by need differences. Black individuals with schizophrenia may have more severe psychiatric illness than Whites because social disadvantage can affect treatment access and effectiveness (Bresnahan et al., 2007; Earl et al., 2011; Morgan & Hutchinson, 2010); consequently, their higher utilization of high-intensity BHO care may be warranted. Similarly, Whites’ higher odds of suicide-related admissions may be warranted as White race appears to be a risk factor for suicide attempts among individuals with schizophrenia (Cassidy et al., 2017). The racial and ethnic differences in cardiometabolic admissions may partly reflect underlying population differences in risk for cardiometabolic disorders, higher for Black individuals relative to Whites (U.S. Department of Health & Human Services Office of Minority Health, 2019, U.S. Department of Health & Human Services Office of Minority Health, 2020). However, Latinx (Daviglus et al., 2012, 2014) and Asian (Jose et al., 2014) individuals are also at higher risk for these conditions than Whites, yet their likelihood of cardiometabolic admissions was at best comparable to that of Whites. Moreover, the emergence in the post-pandemic surge period of a lower likelihood of pneumonia admissions for Black and Latinx beneficiaries relative to Whites is inconsistent with evidence that in New York State and elsewhere, the pandemic affected racial and ethnic minorities more severely (Chowkwanyun & Reed, 2020; NYC Health, 2020; Webb Hooper et al., 2020).

Multiple factors may have caused changes in service utilization immediately after the onset of the COVID-19 pandemic (Hamada & Fan, 2020; Melamed et al., 2020). These include healthcare disruptions caused by reduced in-person services, variable speed of adoption of telehealth, and clinician shortages; reduced patient mobility because of the lockdown and other policies aimed at reducing viral spread; and patients’ new onset homelessness or increased financial strain. However, limited evidence suggests that BHO utilization did not drop among individuals with schizophrenia following the pandemic onset, in part from their robust uptake of telehealth (Lynch et al., 2020; Yang et al., 2020). On the other hand, evidence that disadvantaged populations have less reliable access to telehealth-enabling technology (smartphones, computers, the Internet) (Raja et al., 2021; Uscher-Pines et al., 2020) suggests that among individuals with schizophrenia, racial and ethnic minority groups may have had lower telehealth adoption than Whites.

### Implications of Our Findings

Despite concerns about access to healthcare during the COVID-19 pandemic for socially vulnerable populations with chronic conditions, our findings indicate that Medicaid-funded health services were largely preserved in New York State for individuals with schizophrenia. It is particularly noteworthy that the utilization of ACT and other intensive outpatient services increased, suggesting that providers were able to increase access to address a real or perceived increase in the need for these services stemming from the crisis.

Access to BHO services for Medicaid beneficiaries was sustained under emergency state and federal regulations that facilitated use of video as well as telephone-based telehealth services and state policies that facilitated access to pharmacy services (Center for Connected Health Policy, 2020; Goldman et al., 2020; New York State Department of Health, 2020; New York State Office of Mental Health, 2020). State emergency regulations waived requirements for face-to-face encounters for ACT teams and increased flexibility for clinical documentation and duration of contacts, making it possible for providers to continue to offer services and meet billing criteria. Additionally, several strategies aimed at fostering rapid inpatient bed expansion may have contributed to our finding that access to non-COVID related inpatient services for life-threatening emergencies was largely preserved.

Although our study was not designed to test the causal effects of these interventions on access to healthcare, our findings suggest that the policy response was largely effective for a population for whom unfettered access is vital. A notable achievement is that for the most part, pre-existing racial and ethnic differences were not widened despite the radical and abrupt change in methods of service delivery and pressure on inpatient beds precipitated by the pandemic.

Access to inpatient services for pneumonia at a time of competition for scarce resources including beds and ventilators was a concerning exception—the emergence of a racial and ethnic difference contradicted expectations in a state where the COVID-19 disease burden was higher for minorities.

### Limitations

Our study has some limitations. First, we only adjusted for SSI as a disability indicator and thus, some of the observed racial and ethnic access differences may reflect need differences. We did not adjust for other health status measures because to do so, we would need a longer period of continuous enrollment preceding the utilization and this would preclude a clear assessment of the state’s healthcare system’s COVID-19 response capacity. Second, we did not adjust for clustering of individuals within counties due to the combination of low event rates, county size, and unit of time. The SEs of the estimated regression coefficients are therefore smaller than they should be. Third, some of our effects may be confounded by geographic differences given the higher proportion of Black and Latinx individuals in New York City, the epicenter of the pandemic during the study period, for which our analyses may not have fully controlled. Last, our study may not be generalizable to other states.

## Conclusions

Individuals with schizophrenia, particularly racial and ethnic minorities, are at high risk for poor outcomes during public health emergencies such as the ongoing COVID-19 pandemic. This risk is not only COVID-19-related as access disruptions may have deleterious effects on other health domains. Although our study was not designed to assess causality, our findings suggest that policies implemented by New York State including those that facilitated use of telehealth and pharmacy services largely succeeded at preserving healthcare access for Medicaid beneficiaries. However, there may be a need for policies aimed at preventing unwarranted racial and ethnic differences in access to life-preserving treatment for the driver of the public health emergency. Future research should focus on identifying strategies to ensure equitable access to scarce resources.

## Supplementary Information

Below is the link to the electronic supplementary material.

Supplementary file1 (DOCX 881 kb)
