# Correction to: Healthcare Access for a Diverse Population with Schizophrenia Following the Onset of the COVID‑19 Pandemic

**DOI:** 10.1007/s10597-023-01150-w

**Published:** 2023-06-13

**Authors:** Marcela Horvitz‑Lennon, Emily Leckman‑Westin, Molly Finnerty, Junghye Jeong, Jeannette Tsuei, Katya Zelevinsky, Qingxian Chen, Sharon‑Lise T. Normand

**Affiliations:** 1 RAND Corporation, 20 Park Plaza, Suite 920, Boston, MA 02116 USA; 2 Department of Psychiatry, Cambridge Health Alliance, Harvard Medical School, 1493 Cambridge Street, Cambridge, MA 02139 USA; 3 Department of Health, Office of Mental Health, 44 Holland Avenue, Albany, New York State, NY 12229 USA; 4 Department of Epidemiology and Biostatistics, School of Public Health, University at Albany, State University of New York, 1 University Pl, Rensselaer, NY 12144 USA; 5 RAND Corporation, 1776 Main Street, Santa Monica, CA 90407 USA; 6 Department of Health Care Policy, Harvard Medical School, 180 Longwood Avenue, Boston, MA 02115 USA; 7 Department of Biostatistics, Harvard School of Public Health, 677 Huntington Ave, Boston, MA 02115 USA

## Correction to

Community Mental Health Journal

10.1007/s10597-023-01105-1

The original version of this article unfortunately contains error in Funding section. The part of funding information is missing in the article.

The complete Funding section is given below:

**Funding** Open access funding provided by SCELC, Statewide California Electronic Library Consortium. This publication was made possible by Grant numbers R01MD012428 and R01MD012428-04S1 from the National Institute on Minority Health and Health Disparities. Its contents are solely the responsibility of the authors and do not necessarily represent the official views of the National Institute on Minority Health and Health Disparities.

The original article has been corrected.

