# Supplementary material for: Healthcare Access for a Diverse Population with Schizophrenia Following the Onset of the COVID-19 Pandemic
Source: Community Ment Health J. 2023 May 18;60(1):72–80. doi: 10.1007/s10597-023-01105-1 (PMC10193305; doi:10.1007/s10597-023-01105-1)
Supplement: Supplementary file 1 — Supplementary file1 (DOCX 881 kb) [file 10597_2023_1105_MOESM1_ESM.docx]

**Supplementary Information**

**Analytic Plan**

Data source

We used data from the Medicaid Data Warehouse maintained by the Office of Mental Health (OMH), New York State’s mental health authority, containing information on eligibility, demographic characteristics, diagnoses, and service and pharmacy utilization for Medicaid beneficiaries with behavioral health needs. Individuals are identified as having behavioral health needs if they have received a behavioral health diagnosis, service, or medication. All Medicaid-paid records are available, regardless of whether their care is paid under fee-for-service or managed care arrangements. The OMH Medicaid Data Warehouse is maintained by the state’s Information Technology Services and updated on a weekly basis with data from the Medicaid Data Warehouse maintained by the state’s Medicaid agency (Department of Health).

Variables

Outcome variables

We constructed six binary outcome measures selected based on their importance in the treatment of schizophrenia or comorbid conditions. If a beneficiary utilized the same service more than once during the unit of time in which the measure was assessed, we only counted it once.

*Routine behavioral health outpatient care* captured daily utilization of clinic-based or telehealth services that should be standard care for this population. Services included all care delivered in OMH-licensed clinics or by non-licensed individual practitioners conditional on the observation of a primary behavioral health diagnosis or service (e.g., family psychotherapy).

*High-intensity behavioral health outpatient care* captured monthly utilization of any of three team-based specialty services: Assertive Community Treatment (ACT); Personalized Recovery Oriented Services (PROS), a psychosocial rehabilitation program that integrates rehabilitation, treatment, and support services for people with serious mental illness; an­­d behavioral health Home and Community-Based Services (HCBS) including individual employment support, education support, peer services, and crisis services. These services were assessed monthly because the state pays for them using bundled monthly rates reflecting varying intensity of program utilization. Individuals met this composite measure if any of the respective monthly rates was observed; however, we also report on unadjusted results for each of the specialty services (ACT, PROS, and behavioral health HCBS).

*Antipsychotic drug prescription fills* captured daily observation of antipsychotic drug prescriptions filled at retail pharmacies regardless of quantity supplied (e.g., 30 or 90 days); injectable drugs were also captured if filled at retail pharmacies or if the injection was provided at a clinic/outpatient visit.

*Psychiatric admissions for suicidality* (suicide-related admissions) captured daily admissions prompted by suicidality. We defined suicidality as nonfatal suicide attempt and intentional self-harm and ascertained it using the Center for Disease Control’s algorithm (Hedegaard et al., 2018), as used in other research (Kammer, Rahman, & Chen, 2018). Individuals met this measure if suicidality was listed among the admission diagnoses.

| *Diabetes Mellitus & Cardiovascular Disorders ICD-10 codes* | |
| --- | --- |
| *Diabetes Mellitus* | *Cardiovascular Disorders* |
| E08.01 – E08.11 | A52.19 |
| E09.01 – E09.11 | I21.09, I21.11 – I21.19, I21.29 – I21.4 |
| E09.641 | I25.42 |
| E10.1 – E10.11 | I61.9 |
| E10.641 | I63.019, I63.119, I63.139, I63.20, I63.219 – I63.22, I63.239, I63.30, I63.40, I63.50, I63.59 |
| E11.00 – E11.11 | I65.09 – I65.1, I65.29 |
| E11.641 | I66.09, I66.19, I66.29, I66.9 |
| E13.01 – E13.11 | I71.00 – I71.1, I71.3, I71.5, I71.8 |
| E13.641 | I77.71 – I77.74, I77.79 |

| *Pneumonia ICD-10 codes* |
| --- |
| J09 – J09.X9 |
| J10 – J10.89 |
| J11 – J11.89 |
| J12 – J12.9 |
| J13 |
| J14 |
| J15 – J15.9 |
| J16 – J16.8 |
| J17 |
| J18 – J18.9 |

*Admissions for cardiometabolic emergencies* (cardiometabolic admissions) captured daily admissions for life-threatening emergencies (Lange et al., 2020) associated with cardiovascular disorders (CVD), hereafter CVD admissions (e.g., acute myocardial infarction, intracerebral hemorrhage) or diabetes mellitus, hereafter diabetes admissions (e.g., uncontrolled diabetes with ketoacidosis or hyperosmolarity) (see ICD-10 codes in Box). Individuals met this composite measure if any of the respective ICD-10 codes was listed among the admission diagnoses; however, we also report on unadjusted results for individual measures (CVD admissions and diabetes admissions).

*Pneumonia admissions* captured daily admissions for pneumonia, a seasonal condition that is most prevalent during cold weather months (pneumonia season as defined in the Study cohort and data source section). We defined pneumonia using ICD-10 codes (see Box) indicating any viral or bacterial pneumonia, including codes suggestive or indicative of COVID-19 pneumonia first used in the state in March 2020. Individuals met this measure if any of the ICD-10 codes was listed among the admission diagnoses.

Independent variables

We constructed several person-level variables assessed at the beneficiary’s index eligibility date unless otherwise indicated. The main independent variables of interest were race/ethnicity, time, pandemic period, and their interaction.

*Race/ethnicity* was categorized as White (reference category), non-Latino Black, Latinx, Asian/Other, with the Other category mainly corresponding to American-Indian, and unknown race/ethnicity, as assessed at the index eligibility date. We reduced the proportion of beneficiaries with unknown race/ethnicity using an algorithm (Horvitz-Lennon et al., 2014) that reclassifies race/ethnicity using information captured in the following year for beneficiaries observed in more than one year. Medicaid agencies collect self-reported race/ethnicity data through a paper or online application during the eligibility determination and enrollment processes, with additional data collected during the renewal or redetermination processes in some states including New York State.

*Time* was operationalized as days or months as a count starting with -366 (or -13) for the initiation of the pre-pandemic period, March 7, 2019 set to day/month = 0, and ending at day (or month) 70 (2).

*Pandemic period* was set to 0 in the pre-pandemic period and 1 in the post-pandemic period.

*Other demographic variables* included sex (binary male/female, with male as reference group) and age (categorical: 18-24, 25-44, 45-64, with 45-64 as reference group).

*Medicaid eligibility mechanism* was set to one if the beneficiary ever had Medicaid eligibility through Supplemental Security Income (SSI) during the study period and 0 otherwise, with SSI as the reference category. The variable was used as a health status indicator given that SSI eligibility requires demonstration of disability.

*County of residence* described beneficiaries’ place of residence (any out of 62 counties, with New York City as reference county).

Statistical models

For each outcome, we estimated a set of nested logistic regression models that differed in terms of the type of interactions included. All models included sex (female), SSI, age groups, pandemic period, and county (see Table with estimated regression coefficients in next Online section). Race/ethnicity, time (day or month), county, and their pair-wise interactions were also included. We compared models using the Akaike Information Criterion (AIC) and selected the model with the smallest value AIC value. If the difference between a model AIC and the minimum AIC was < 2 (implying two models are competitive), we selected the model with the fewest predictors to be parsimonious. The “largest” model we estimated was:

$$logit\left( p \right)=b_{0}+b_{1}day+b_{2}day^{2}+b_{3}post+b_{4}\left( post\times day \right)+b_{5}Black+b_{6}\left( Black\times post \right)+b_{7}\left( Black\times day \right)+ b_{8}Latinx+b_{9}\left( Latinx\times post \right)+b_{10}\left( Latinx\times day \right)+ b_{11}Asian/Other+b_{12}\left( Asian/Other\times post \right)+b_{13}\left( Asian/Other\times day \right)+ b_{14}\left( Unknown Race/Ethnicity \right)+b_{15}\left( Unknown Race/Ethnicity\times post \right)+b_{16}\left( Unknown Race/Ethnicity\times day \right)+ b_{17}county+\boldsymbol{b}_{18}\left( county\times day \right)+b_{19}female+b_{20}SSI+b_{21}(age groups)$$

where

| Variable | Definition |
| --- | --- |
| P (Outcome) | Pr(y = 1) where y=1 for occurrence of the outcome and 0 otherwise |
| Day | (t-366)/100 where t is a count from 0 (corresponding to March 7, 2019) to the last day of the study period. For models that used months, month was a count from 1 to 13 and we used (t-13) |
| Post | Binary variable = 1 if day of observation is after March 7, 2020 and 0 otherwise |
| Black, Latinx, Asian/Other,  Unknown | Each binary-valued race/ethnicity variable was centered at the daily county mean. For example, letting $Black_{ijt}=1$ if the j^th^ observation on day *t* in the i^th^ county corresponded to a Black beneficiary and 0 otherwise, $n_{it}$ denotes the number of all eligible beneficiaries on day *t* in the i^th^ county,  $\bar{Black}_{it}=\frac{1}{n_{it}}\sum_{j} Black_{ijt}$ then the race/ethnicity variable included in the model was ${Black_{ijt}- \bar{Black}}_{it}$. The reference category is White race. |
| Age groups: | Age was grouped into 18-24 years and 25-44 years, and coded as two binary variables, coded 1 if the beneficiary fell into the age group and 0 otherwise. The reference group is 45-64 years. |
| Female | Binary variable = 1 if beneficiary is female. The reference group is male. |
| SSI | Binary variable = 1 if the beneficiary ever had Medicaid eligibility through Supplemental Security Income (SSI) during the study period; 0 otherwise. The reference category is SSI. |
| County | A set of binary variables for each county. The reference county is New York City |

Supplementary References

Hedegaard, H., Schoenbaum, M., Claassen, C., Crosby, A., Holland, K., & Proescholdbell, S. (2018). Issues in Developing a Surveillance Case Definition for Nonfatal Suicide Attempt and Intentional Self-harm Using International Classification of Diseases, Tenth Revision, Clinical Modification (ICD-10-CM) Coded Data. *Natl Health Stat Report*(108), 1-19. Retrieved from <http://www.ncbi.nlm.nih.gov/pubmed/29616901>

Horvitz-Lennon, M., Volya, R., Donohue, J. M., Lave, J. R., Stein, B. D., & Normand, S.-L. T. (2014). Disparities in Quality of Care among Publicly Insured Adults with Schizophrenia in Four Large US States, 2002-2008. *Health Services Research, 49*(4), 1121-1144. doi:10.1111/1475-6773.12162

Kammer, J., Rahman, M., & Chen, Q. (2018). Creating suicide attempt/intentional self-harm episodes using administrative billing data. 1-7. Retrieved from <https://www.lexjansen.com/mwsug/2018/HS/MWSUG-2018-HS-53.pdf>

Lange, S. J., Ritchey, M. D., Goodman, A. B., Dias, T., Twentyman, E., Fuld, J., . . . Yang, Q. (2020). Potential Indirect Effects of the COVID-19 Pandemic on Use of Emergency Departments for Acute Life-Threatening Conditions - United States, January-May 2020. *MMWR Morb Mortal Wkly Rep, 69*(25), 795-800. doi:10.15585/mmwr.mm6925e2

| **Supplementary Table 1. Estimated regression coefficients (logit scale), by outcome** (N=27,359,993 person-days). *Measured monthly (N=887,910 person-months); **Coefficient for 25-44 age group. ^¶^Centered at county mean. N/A = not applicable because the variable was not selected for inclusion in final model. | | | | | | |
| --- | --- | --- | --- | --- | --- | --- |
| **Coefficient** | **Utilization Outcome: Estimate (SE)** | | | | | |
|  | **Behavioral Health Care** | | **Antipsychotic drug prescription fills** | **Suicide-related admissions** | **Cardiometabolic admissions** | **Pneumonia admissions** |
|  | **Routine** | **High-intensity*** |  |  |  |  |
| Intercept | -3.40 (0.01) | -2.89 (0.03) | -3.72 (0.01) | -10.25 (0.14) | -10.54 (0.14) | -85.87 (6.79) |
| Day (Month) | 0.33 (0.01) | 0.03 (0.01) | 0.08 (0.01) | 0.08 (0.27) | -0.33 (0.24) | -39.80 (3.58) |
| Day^2^ | 0.02 (0.00) | N/A | 0.01 (0.00) | N/A | N/A | -13.38 (1.19) |
| Post-Pandemic | -0.10 (0.00) | 0.02 (0.01) | -0.02 (0.00) | -0.07 (0.12) | 0.04 (0.10) | 73.44 (6.50) |
| Day(Month) | 0.28 (0.01) | 0.01 (0.01) | 0.02 (0.01) | 0.14 (0.27) | -0.29 (0.24) | 48.74 (4.34) |
| Black^¶^ | -0.28 (0.00) | 0.19 (0.02) | -0.24 (0.00) | -0.57 (0.11) | 0.30 (0.10) | -0.04 (0.13) |
| × Post | 0.02 (0.01) | 0.00 (0.02) | 0.00 (0.00) | N/A | N/A | -0.19 (0.13) |
| × Day | -0.02 (0.00) | N/A | N/A | N/A | N/A | N/A |
| Latinx^¶^ | -0.12 (0.01) | -0.10 (0.02) | -0.14 (0.01) | -0.37 (0.13) | -0.06 (0.12) | -0.12 (0.15) |
| × Post | 0.00 (0.01) | -0.01 (0.02) | 0.00 (0.01) | N/A | N/A | -0.27 (0.15) |
| × Day | -0.01 (0.00) | N/A | N/A | N/A | N/A | N/A |
| Asian/Other^¶^ | -0.08 (0.01) | 0.00 (0.03) | 0.07 (0.01) | -0.69 (0.21) | -1.29 (0.28) | -1.58 (0.59) |
| × Post | -0.02 (0.01) | -0.02 (0.03) | -0.02 (0.01) | N/A | N/A | 0.92 (0.59) |
| × Day | -0.01 (0.00) | N/A | N/A | N/A | N/A | N/A |
| Unknown^¶^ | -0.14 (0.01) | -0.37 (0.02) | -0.09 (0.01) | -0.40 (0.15) | -0.35 (0.16) | -0.68 (0.28) |
| × Post | 0.01 (0.01) | 0.02 (0.03) | 0.00 (0.01) | N/A | N/A | 0.46 (0.28) |
| × Day | -0.01 (0.00) | N/A | N/A | N/A | N/A | N/A |
| Female | 0.06 (0.00) | -0.05 (0.00) | 0.02 (0.00) | 0.10 (0.04) | -0.10 (0.04) | 0.04 (0.04) |
| SSI | -0.12 (0.00) | -0.54 (0.00) | -0.28 (0.00) | 0.08 (0.04) | 0.12 (0.04) | -0.02 (0.04) |
| Age Groups** | 0.01 (0.00) | 0.24 (0.01) | 0.03 (0.00) | 0.05 (0.06) | -0.17 (0.06) | -0.09 (0.09) |
| Fit Statistics | | | | | | |
| **Source:** Authors’ analyses of New York State Medicaid data (March 7, 2019-May 15, 2020) | | | | | | |

**Supplementary Tables**

| **Supplementary Figure 1. Distribution of County/County*Day (Month) Effects versus New York City, by outcome.** Some confidence intervals are wide given the small sample size at the county-level. |
| --- |
| **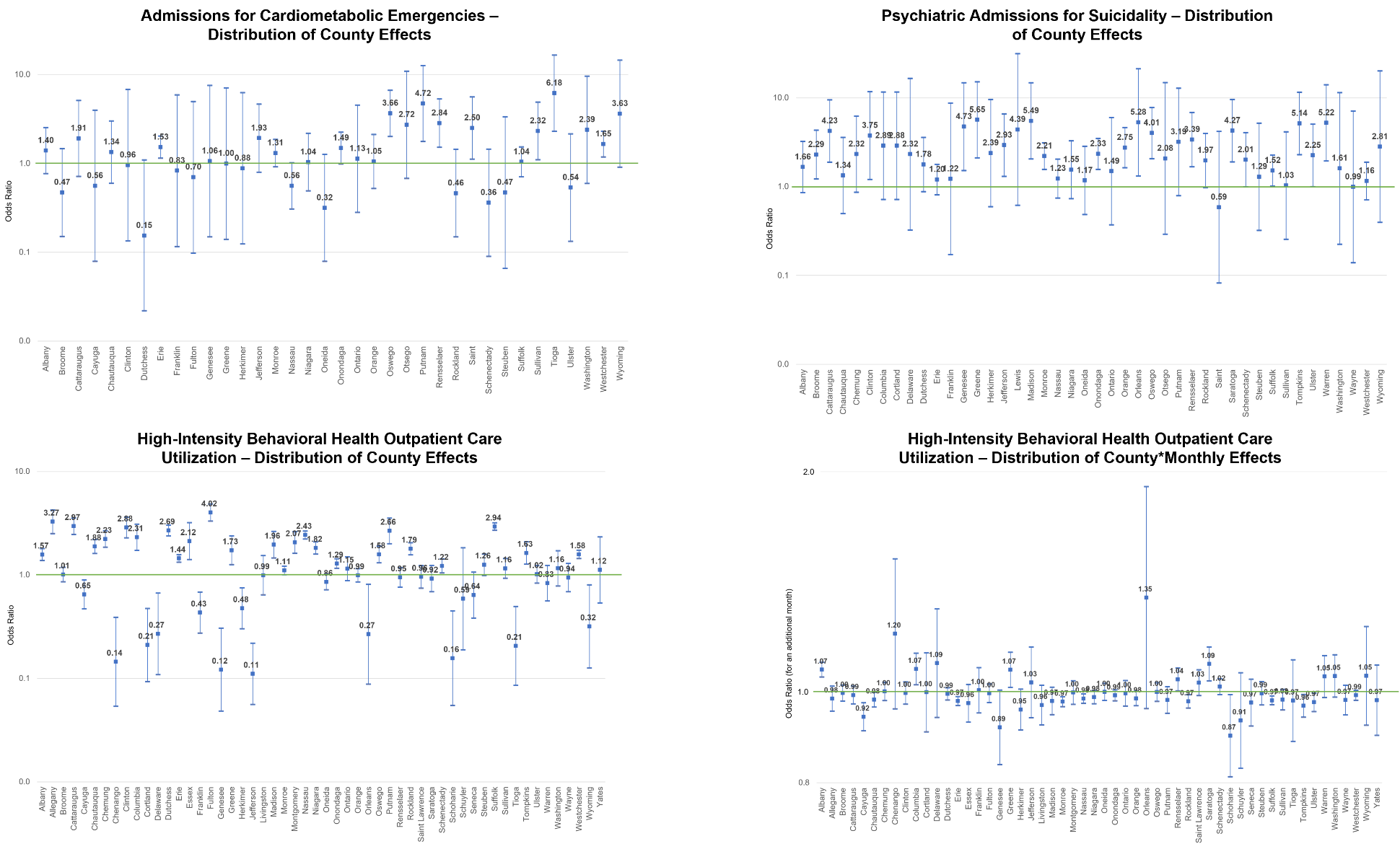**  **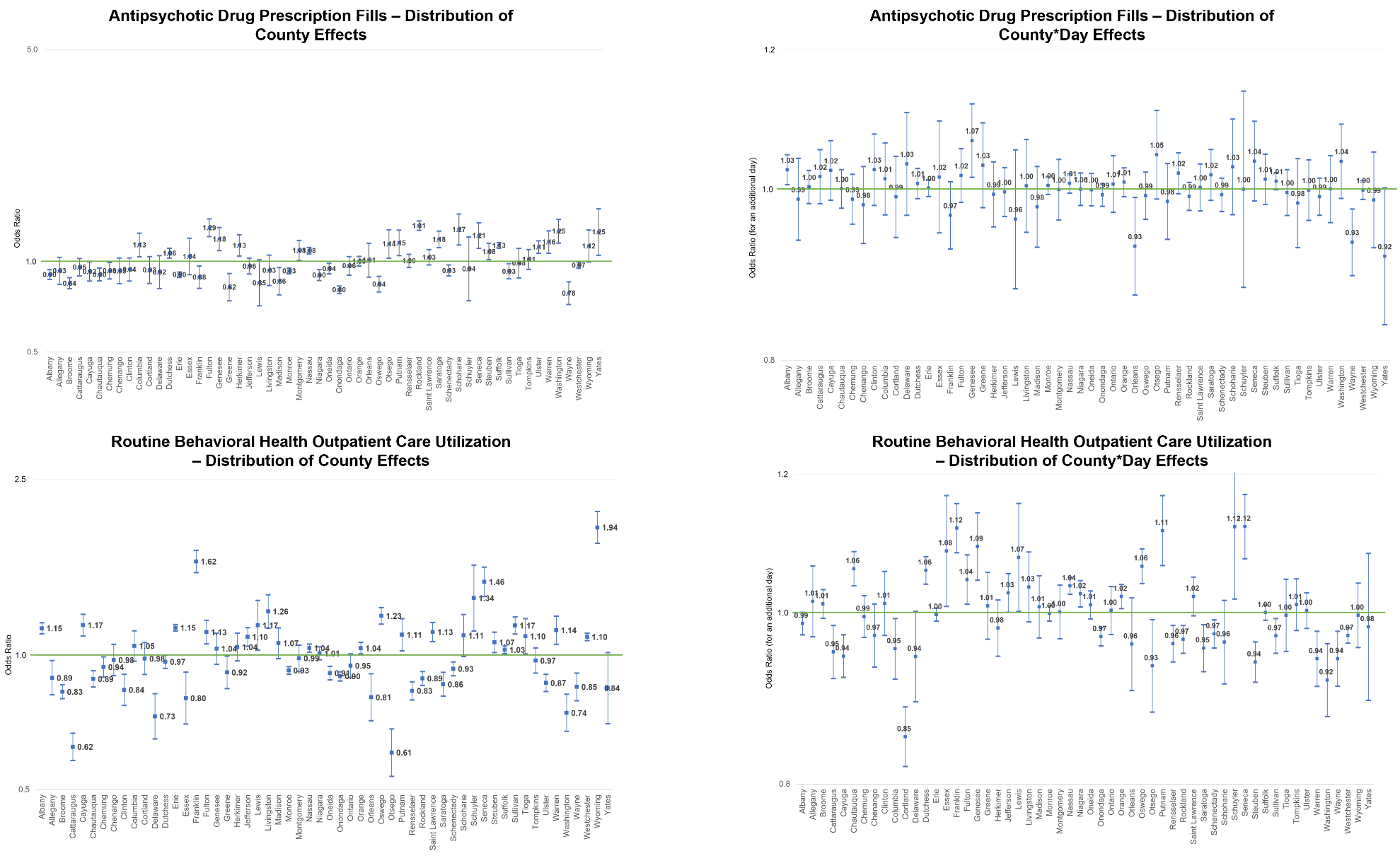**  **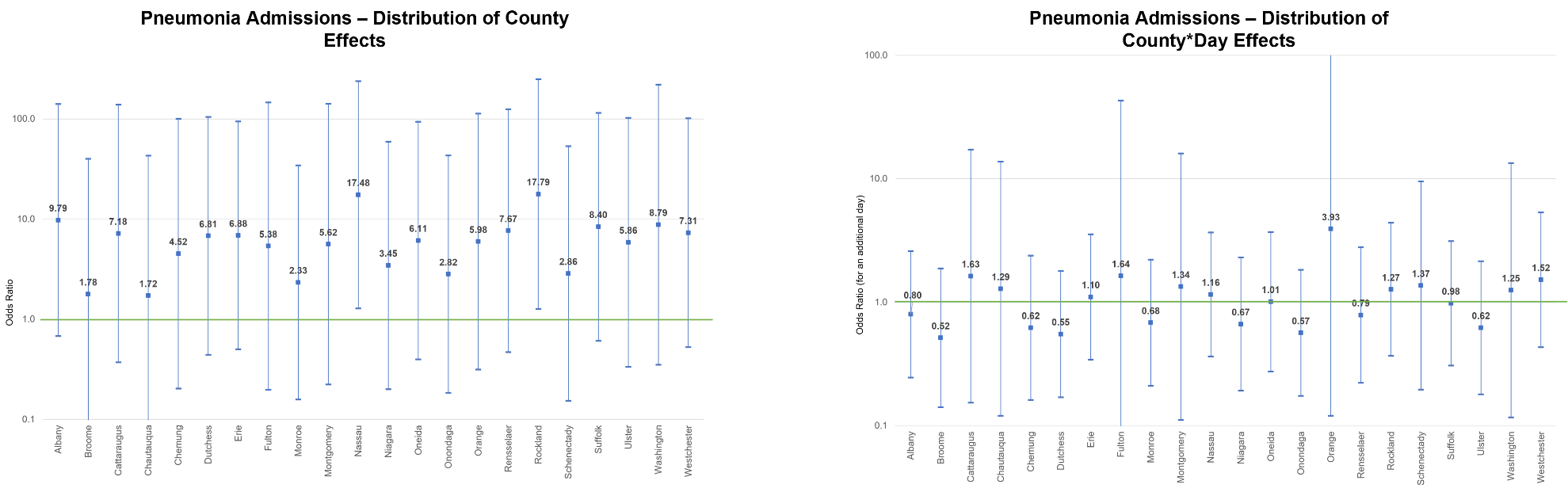** |
| **Source:** Authors’ analysis of New York State Medicaid data (March 7, 2019-May 15, 2020) |

| **Supplementary Table 2. Characteristics of beneficiaries included compared to those excluded due to lacking county information**, by period. | | | | |
| --- | --- | --- | --- | --- |
| **Characteristic** | **Included** | | **Excluded** | |
|  | **Persons** | **Person-Days** | **Persons** | **Person-Days** |
| **Pre-pandemic: March 7, 2019 – March 6, 2020** | | | | |
| Number | 67,184 | 22,976,921 | 988 | 273,904 |
| Female, n (%) | 26,173 (39.0%) | 9,041,810 (39.4%) | 266 (26.9%) | 74,033 (27.0%) |
| Age Group, n (%) | | | | |
| 18-24 | 6,863 (10.2%) | 2,277,333 (9.9%) | 89 (9.0%) | 15,030 (5.5%) |
| 25-44 | 30,174 (44.9%) | 10,158,267 (44.2%) | 343 (34.7%) | 90,067 (32.9%) |
| 45-64 | 30,147 (44.9%) | 10,541,321 (45.9%) | 556 (56.3%) | 168,807 (61.6%) |
| Race/Ethnicity, n (%) | | | | |
| Black | 26,112 (38.9%) | 8,971,082 (39.0%) | 485 (49.1%) | 135,740 (49.6%) |
| Latinx | 12,694 (18.9%) | 4,487,750 (19.5%) | 208 (21.1%) | 59,654 (21.8%) |
| White | 16,399 (24.4%) | 5,645,007 (24.6%) | 190 (19.2%) | 53,898 (19.7%) |
| Asian/Other | 4,545 (6.8%) | 1,543,980 (6.7%) | 65 (6.6%) | 16,225 (5.9%) |
| Unknown | 7,434 (11.1%) | 2,329,102 (10.1%) | 40 (4.0%) | 8,387 (3.1%) |
| SSI, n (%) | 38,075 (56.7%) | 13,632,584 (59.3%) | 444 (44.9%) | 141,016 (51.5%) |
| **Post-pandemic: March 7, 2020 – May 15, 2020** | | | | |
| Number | 63,369 | 4,383,072 | 799 | 52,965 |
| Female, n (%) | 24,818 (39.2%) | 1,719,169 (39.2%) | 209 (26.2%) | 13,730 (25.9%) |
| Age Group, n (%) | | | | |
| 18-24 | 6,354 (10.0%) | 438,705 (10.0%) | 35 (4.4%) | 2,107 (4.0%) |
| 25-44 | 28,283 (44.6%) | 1,955,283 (44.6%) | 275 (34.4%) | 18,064 (34.1%) |
| 45-64 | 28,732 (45.3%) | 1,989,084 (45.4%) | 489 (61.2%) | 32,794 (61.9%) |
| Race/Ethnicity, n (%) | | | | |
| Black | 24,674 (38.9%) | 1,706,607 (38.9%) | 398 (49.8%) | 26,036 (49.2%) |
| Latinx | 12,237 (19.3%) | 848,332 (19.4%) | 177 (22.2%) | 11,940 (22.5%) |
| White | 15,523 (24.5%) | 1,075,964 (24.5%) | 151 (18.9%) | 10,059 (19.0%) |
| Asian/Other | 4,270 (6.7%) | 296,123 (6.8%) | 49 (6.1%) | 3,339 (6.3%) |
| Unknown | 6,665 (10.5%) | 456,046 (10.4%) | 24 (3.0%) | 1,591 (3.0%) |
| SSI, n (%) | 36,271 (57.2%) | 2,523,092 (57.6%) | 373 (46.7%) | 25,598 (48.3%) |
| **Source:** Authors’ analyses of New York State Medicaid data (March 7, 2019-May 15, 2020) | | | | |

| **Supplementary Table 3. Unadjusted utilization rates.** Mean and Standard Error (SE), per 100,000 Person-Days unless indicated, Pre- and Post-Pandemic Periods, and their Differences (Post-Pre-Pandemic Difference), expressed as Mean and 95% Confidence Interval (CI). | | | |
| --- | --- | --- | --- |
| **Outcome Measure** | **Pre-Pandemic**  **3/7/19–3/6/20**  **Mean (SE)** | **Post-Pandemic**  **3/7-5/15/2020**  **Mean (SE)** | **(Post)–(Pre) Pandemic Difference**  **Mean (95% CI)** |
| Routine behavioral health outpatient care, daily | 3411.9 (105.4) | 3528.5 (242.3) | 116.6 (-400.8, 634.0) |
| High-intensity behavioral health outpatient care, monthly | 7737.5 (59.9) | 8386.8 (107.8) | 649.3 (310.8, 987.8) |
| Assertive Community Treatment, monthly | 3748.3 (34.1) | 4100.0 (72.0) | 351.7 (157.1, 546.3) |
| Personalized Recovery Oriented Services, monthly | 3209.8 (18.6) | 3231.6 (10.0) | 21.7 (-81.3, 124.8) |
| Behavioral health Home and Community-Based Services, monthly | 858.2 (31.7) | 1146.0 (22.1) | 287.8 (112.4, 463.2) |
| Antipsychotic drug prescription fills, daily | 2336.8 (51.7) | 2401.1 (118.2) | 64.4 (-189.1, 317.8) |
| Suicide-related admissions | 2.3 (0.1) | 1.9 (0.2) | -0.4 (-0.8, 0.1) |
| Cardiometabolic admissions | 3.0 (0.1) | 2.5 (0.2) | -0.5 (-1.1, 0.0) |
| Cardiovascular disorders admissions | 0.9 (0.1) | 0.7 (0.1) | -0.2 (-0.5, 0.1) |
| Diabetes admissions | 2.1 (0.1) | 1.8 (0.2) | -0.3 (-0.8, 0.1) |
| Pneumonia admissions | 3.3 (0.3) | 15.5 (1.1) | 12.2 (9.8, 14.5) |
| **Note:** Pneumonia season (pre-pandemic period = 3/7– 5/15/2019; post-pandemic period = 3/7 -5/15/2020). Means calculated as the (average daily number of services)/(average daily number of eligible person-days) multiplied by 100,000. The pre-pandemic period averaged over 366 days while the post-pandemic period averaged over 70 days.  **Source:** Authors’ analyses of New York State Medicaid data (March 7, 2019-May 15, 2020). | | | |
